# Secreted Phosphoprotein 1 and Sex-Specific Differences in Silica-Induced Pulmonary Fibrosis in Mice

**DOI:** 10.1289/ehp.1510335

**Published:** 2016-03-08

**Authors:** Joseph D. Latoche, Alexander Chukwuma Ufelle, Fabrizio Fazzi, Koustav Ganguly, George D. Leikauf, Cheryl L. Fattman

**Affiliations:** 1 Department of Environmental and Occupational Health, Graduate School of Public Health, University of Pittsburgh, Pittsburgh, Pennsylvania, USA; 2 SRM (Sri Ramaswamy Memorial) Research Institute, SRM University, Chennai, India

## Abstract

**Background:**

Fibrotic lung diseases occur predominantly in males, and reports describe better survival in affected females. Male mice are more sensitive to silica-induced lung fibrosis than silica-treated female mice. Secreted phosphoprotein 1 (SPP1, also known as osteopontin) increases in pulmonary fibrosis, and Spp1 transcription may be regulated by estrogen or estrogen receptor–related receptors.

**Objective:**

We determined whether differences in silica-induced SPP1 levels contribute to sex differences in lung fibrosis.

**Methods:**

Male and female mice were treated with 0.2 g/kg intratracheal silica, and lung injury was assessed 1, 3, or 14 days post-exposure. Gene-targeted (Spp1–/–) mice, control Spp1+/+ (C57BL/6J) mice, ovariectomized (OVX) female mice, and estrogen-treated male mice were treated with silica, and lung injury was assessed.

**Results:**

Silica-induced SPP1 in lung tissue, bronchoalveolar lavage, and serum increased more in male than in female mice. Following silica treatment, bronchoalveolar lavage cell infiltrates decreased in female Spp1–/– mice compared with female Spp1+/+ mice, and lung hydroxyproline decreased in male Spp1–/– mice compared with male Spp1+/+ mice. OVX female mice had increased lung SPP1 expression in response to silica compared with silica-treated sham female mice. Silica-induced lung collagen and hydroxyproline (markers of fibrosis), and SPP1 levels decreased in estrogen-treated males compared with untreated males.

**Conclusion:**

These findings suggest that sex-specific differences in SPP1 levels contribute to the differential sensitivity of male and female mice to the development of silica-induced fibrosis.

**Citation:**

Latoche JD, Ufelle AC, Fazzi F, Ganguly K, Leikauf GD, Fattman CL. 2016. Secreted phosphoprotein 1 and sex-specific differences in silica-induced pulmonary fibrosis in mice. Environ Health Perspect 124:1199–1207; http://dx.doi.org/10.1289/ehp.1510335

## Introduction

A chronic fibrotic lung disease caused by silica inhalation, silicosis is a detrimental occupational disease, with thousands of new cases being reported worldwide every year (Leung et al. 2012). Hazardous occupational exposures occur in mining, sandblasting, road construction, pottery making, masonry, and tunneling operations. Recently, silica exposures have occurred during hydraulic fracturing of gas and oil wells (Esswein et al. 2013) and during the fabrication and installation of engineered stone countertops (Kramer et al. 2012). Nonoccupational silica exposures can result near industrial and nonindustrial sources (Bhagia 2012). In the United States, age-adjusted mortality rates have dropped from 8.9 per 1,000,000 in 1968 to 0.4 per 1,000,000 in 2010 (Bang et al. 2015). In recent years, silicosis has increased along with increased coal workers’ pneumoconiosis (Halldin et al. 2015; Laney and Weissman 2014) and patients are younger and develop accelerated, severe silicosis with higher mortality (Laney and Weissman 2014).

Most silicosis patients are men because occupations associated with silicosis have been historically male-dominated (Leung et al. 2012), and limited data suggest that survival may be better in women (Morozova 2012). Similarly, other fibrotic lung diseases (e.g., idiopathic pulmonary fibrosis) also occur predominantly in men (Raghu et al. 2006), and women experience better survival (Gribbin et al. 2006; Han et al. 2008; McCormack et al. 1995; Raghu et al. 2014; Schwartz et al. 1994). This discrepancy suggests that sex can influence pulmonary fibrosis pathogenesis. However, the manner in which sex alters the development and progression of pulmonary fibrosis is largely unknown, inasmuch because most studies control for sex rather than examine sex-specific effects. Furthermore, animal studies fail to clearly define the relationship between sex and pulmonary fibrosis (Carey et al. 2007). For example, female rats develop more severe bleomycin-induced pulmonary fibrosis than male rats (Gharaee-Kermani et al. 2005), whereas male mice develop more severe bleomycin-induced pulmonary fibrosis than female mice (Redente et al. 2011; Voltz et al. 2008).

Secreted phosphoprotein-1 (SPP1) is an extracellular matrix protein and cytokine associated with inflammatory and profibrotic effects in a number of organs (Wang and Denhardt 2008). In the lung, macrophages, lymphocytes, and alveolar epithelial cells produce SPP1 (Ganguly et al. 2014; O’Regan 2003). As a pleiotropic cytokine, SPP1 stimulates macrophage and neutrophil chemotaxis, type-1 cytokine secretion, and macrophage and lymphocyte differentiation (Lund et al. 2009; Wang and Denhardt 2008). As a pro-fibrotic molecule, SPP1 controls the expression and augments the effects of other profibrotic mediators, such as transforming growth factor beta 1 (TGFB1), on fibroblast proliferation and myofibroblastic differentiation (Nagao et al. 2012; Xiao et al. 2012). SPP1 also mediates fibroblast migration through integrin- (Anwar et al. 2012; Li et al. 2000) and matrix metalloproteinase–dependent mechanisms (Lund et al. 2009). Lung SPP1 increases in humans with pulmonary fibrosis (Foster et al. 2015; Nau et al. 1997; Pardo et al. 2005) and in mouse (Berman et al. 2004; Miyazaki et al. 1995; Oh et al. 2015; Sabo-Attwood et al. 2011; Takahashi et al. 2001) and rat (Langley et al. 2011; Ma et al. 2012; Mangum et al. 2004) models of pulmonary fibrosis. In addition, SPP1 may be a useful biomarker for the development and progression of fibrotic lung diseases (Boon et al. 2009; Kadota et al. 2005; Kelly et al. 2006; O’Regan et al. 2006; Pardo et al. 2005; Selman et al. 2006; Vij and Noth 2012). Gene-targeted *Spp1^–/–^* mice developed less bleomycin-induced pulmonary fibrosis (Berman et al. 2004) as well as reduced injury in models of renal, heart, kidney, and liver fibrosis (Rittling and Denhardt 1999).

Previously, we observed that silica-treated male mice develop more fibrosis but show a weaker inflammatory response than silica-treated female mice (Brass et al. 2010). Studies of the *SPP1* promoter suggested that estrogen could activate transcription not through classical estrogen response element (ERE) but through estrogen receptor 1 interactions with SF-1 response elements (SFREs) in the promoter (Craig and Denhardt 1991; Vanacker et al. 1999). However, regulation of the *SPP1* promoter is complex and may be tissue- or disease-context dependent. For example, in osteoblasts, *SPP1* also can be activated or repressed by estrogen receptor–related receptors (ESRRs) that also bind SFRE sites in the *SPP1* promoter (Zirngibl et al. 2013). Although these orphan receptors do not bind estrogen, ESRRs can interact with estrogen receptors (Johnston et al. 1997), leaving the role of estrogen in regulating SPP1 uncertain. Thus, this study examined whether altered SPP1 expression is associated with sex difference in silica-induced pulmonary fibrosis in mice.

## Materials and Methods

### Materials

Male and female *Spp1* ^+/+^ (C57BL/6J), ovariectomized (OVX) *Spp1* ^+/+^ (C57BL/6J) female, and *Spp1* ^–/–^ (B6.Cg-Spp1^tm1Blh^/J) (Liaw et al. 1998) mice were obtained from Jackson Laboratories (Bar Harbor, ME). Studies with ovariectomized (OVX) mice were compared to sham mice that had similar surgery without removal of the ovaries (Jackson Laboratories). Studies were conducted with C57BL/6J male mice injected subcutaneously with 250 ng of 17-β estradiol or an equal volume (50 μL) of vehicle (olive oil) daily for 21 days before silica exposure. Silica (Min-U-Sil 5) was provided by A. Ghio [U.S. Environmental Protection Agency (EPA), Durham, NC]. The following chemicals were obtained from identified suppliers: isoflurane (Webster Veterinary, Devens, MA), ketasthesia (Butler-Schein, Dublin, OH), xylazine hydrochloride (MP Biomedicals, Solon, OH), SPP1 enzyme-linked immunosorbent assay (ELISA) and antibody (R&D Systems, Minneapolis, MN), and Masson’s trichrome reagents and hematoxylin (Sigma Chemicals, St. Louis, MO). Immunohistochemistry was performed using secondary antibodies with VECTASTAIN® Elite ABC (Vector Labs, Burlingame, CA) and aminoethyl carbazole substrate (Life Technologies, Camarillo, CA). All other chemicals and reagents were purchased from Thermo Fisher Scientific (Pittsburgh, PA).

### Intratracheal Instillations

Animal procedures were approved by the University of Pittsburgh Institutional Animal Care and Use Committee, and mice used were treated humanely and with regard for alleviation of suffering. Male and female mice were anesthetized briefly with inhaled isoflurane and were given a single intratracheal dose of 0.2 mg/kg Min-U-Sil 5 crystalline silica (median aerodynamic diameter = 2.2 μm) or 0.9% saline in a total volume of 60 μL. Mice were sacrificed 14 days post-exposure.

### Sample Processing

Saline- and silica-treated mice were euthanized at 1, 3, or 14 days post-exposure using 20 mg/kg ketamine and 2 mg/kg xylazine. Bronchoalveolar fluid (BALF) was obtained by instilling and withdrawing 1.3 mL of sterile 0.9% saline. Right lungs were excised and acid hydrolyzed for hydroxyproline determination. Left lungs were either flash-frozen in liquid nitrogen (stored at –80°C) or fixed in 10% buffered formalin (gravity flow at 10 mmHg) and processed for routine histological analysis. At day 14, blood samples were collected via intracardial puncture. Serum was obtained by centrifugation at 2,000 × *g* for 5 min at 20–22°C and stored at –80°C.

### Lavage Total Cell Count, Differential Cell Count and Protein Determination

Total leukocyte cell counts in BALF were determined using a Beckman Dual Z1 Coulter Particle Counter (Coulter, Fullerton, CA). White blood cell differential counts were obtained by staining cytospins of BALF with a Protocol Hema 3 stain (Thermo Fisher Scientific). The percentage and number of total cells and that were macrophages, lymphocytes, and neutrophils were determined using light microscopy. Total BALF protein content was determined using a Coomassie Plus colorimetric assay. Sample absorbance was measured at 595 nm using a Beckman Coulter DU800 spectrophotometer.

### Histochemical Analysis of Formalin-fixed Lung Tissue

Lung sections were stained with Masson’s trichrome, and photomicrographs were captured using a Nikon microscope (Nikon Instruments Inc., Melville, NY) and Nikon Elements analysis software. Lung sections were stained with Masson’s trichrome to visualize collagen. The entire lung was photographed at 20× magnification, and Nikon Elements software was used to calculate tissue volume density (TVD), defined as the percentage of each microscopic field that was lung tissue as well as the percentage of each tissue-containing field that was collagen. Tissue (red) and collagen (blue) staining areas were sampled using six micrographs for each lung as an internal control to account for variability in staining. Values for all fields were averaged to yield a single TVD value per animal. TVD values per animal were then averaged to yield a group average.

Tissue for immunohistochemical analysis was deparaffinized using xylenes and a graded ethanol series. Antigen retrieval was performed by microwaving slides for three consecutive 5-min periods at 20% power in a 1 mM citrate solution, pH 6.0. The slides were rehydrated in PBS, followed by blocking of endogenous peroxidases by immersion in 10% H_2_O_2_ for 10 min. The slides were blocked with 5% horse serum in phosphate-buffered saline (PBS) + 4% bovine serum albumin (BSA) for 30 min at room temperature. Primary antibody omission and *Spp1^–/–^* lung tissue samples were used as negative staining controls. Tissue was stained using a goat anti-SPP1 antibody at 2 μg/mL in 1× PBS + 4% BSA (1 hr, 22°C). Detection of SPP1 was performed using a biotinylated horse–anti-goat secondary antibody at 1:200 dilution, avidin/biotin (VECTASTAIN® Elite ABC), and aminoethyl carbazole substrate. Tissue was counterstained with Mayer’s hematoxylin. Lung SPP1 was quantified by color thresholding analysis using Nikon Elements analysis software.

### SPP1 Analysis in BALF and Serum

An ELISA for SPP1 was performed using the SPP1 ELISA DuoSet (R&D Systems). The absorbance at 450 nm was read using a SpectraMax M2e plate reader (Molecular Dynamics), and standard curves and sample values were generated using SoftMax Pro software.

### Quantitative Reverse Transcriptase–Polymerase Chain Reaction Analysis

Lung tissue was homogenized and total RNA isolated using TRIZOL (Sigma). Isolated RNA was treated with Dnase1 and quantified by A260/A280 spectrophotometric absorbance using a BioTek Synergy 2 multimode microplate reader (BioTek, Winoosk, VT) and Gen5 data analysis software. Two hundred nanograms of RNA was reverse-transcribed using an Iscript cDNA synthesis kit (BioRad) for 5 min at 25°C, 30 min at 42°C, and 5 min at 85°C. One microliter of cDNA was used for each 10-μL quantitative reverse transcriptase–polymerase chain reaction (qRT-PCR) reaction consisting of 5 μL TaqMan Gene Expression Master Mix, 3.5 μL ultrapure water, and 0.5 μL primer for either SPP1 (Mm01611440_mH; Applied Biosystems, Carlsbad, CA) or 18S ribosomal RNA (RPS18) (Mm03928990_g1; Applied Biosystems) as an endogenous control. The reaction cycle was as follows: 2 min at 50°C, 10 min at 95°C, and then 40 cycles of 15 sec at 95°C followed by 1 min at 60°C. Relative levels of SPP1 transcripts were analyzed using the ΔΔ C_T_ method and expressed as log2(fold-change).

### Lung Collagen Content Analysis

Right lungs were excised for hydroxyproline determination via the chloramine-T spectrophotometric absorbance assay as previously described (Fattman et al. 2001; Woessner 1961). Individual tissue samples were placed into 2-mL glass ampules and dried at 110°C. After drying, 2 mL of 6 N HCl was added to each ampule. Oxygen was purged from each ampule and was replaced by nitrogen; then, each ampule was vacuum-sealed and the samples were acid hydrolyzed at 110°C for 24 hr. The acid was completely driven off at 110°C before resuspension of the samples in 2 mL of 1× PBS at 60°C for 1 hr. The rehydrated samples were centrifuged at 20,000 × *g* for 10 min to remove particulate matter.

### Statistical Analysis

All data were evaluated using one-way or two-way analysis of variance (ANOVA) as appropriate and Bonferroni’s post-hoc test for multiple comparisons in each assay. Data with *p*-values ≤ 0.05 were considered significant.

## Results

### SPP1 Expression in Response to Silica Treatment in Male and Female Mice

**Figure 1:**
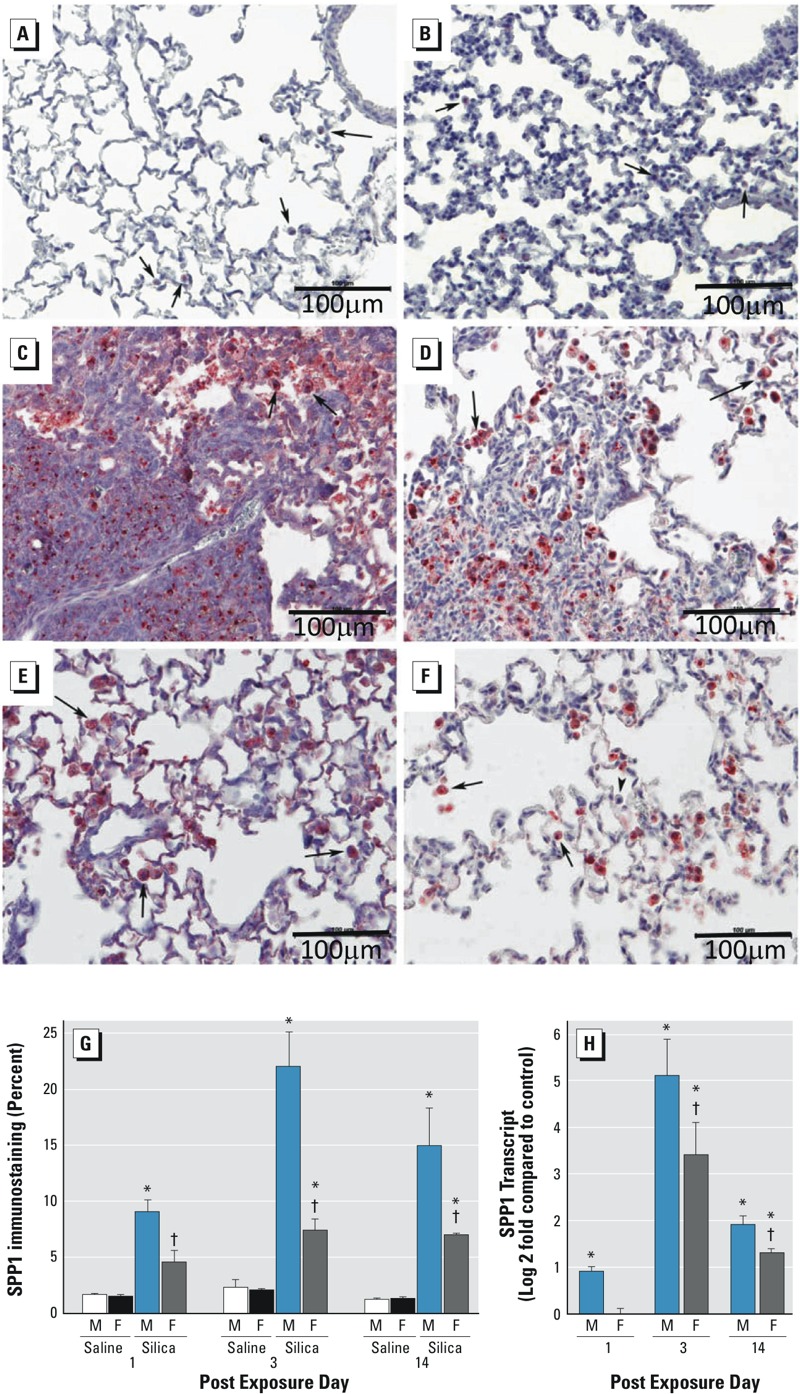
Lung secreted phosphoprotein 1 (SPP1) expression in response to silica treatment in male and female C57BL/6J mice. Immunohistochemical staining for SPP1 in lung tissue from saline-exposed (*A*) male, (*B*) female, and silica-treated (*C* and *E*) male and (*D* and *F*) female C57BL/6J mice at 14 days post-exposure. Panels (*C*)**and (*D*) are representative sections of silica-induced fibrotic lesions in male and female mice, respectively. Panels (*E*) and (*F*) are representative sections of silica-induced SPP1 expression in macrophages (arrows) and alveolar epithelial cells in male and female mice, respectively. (*G*) Percentage of tissue immunostaining for SPP1 protein in C57BL/6J mice at 1, 3, and 14 days post-exposure. (*H*) Lung SPP1 transcripts in silica-treated male and female mice in C57BL/6J mice at 1, 3, and 14 days post-exposure. Transcripts are expressed as log2(fold change) compared with saline-treated, sex-matched controls, and values were normalized to 18S ribosomal protein (RPS18). SPP1 protein immunostaining increased more in male (*C*, *E*, and *G*) than in female (*D*, *F*, and *G*) C57BL/6J mice following silica treatment. Values are means ± SE. Saline-treated groups, *n* = 6 mice/sex/day; silica-treated groups, *n* = 5 mice/sex/day. *p < 0.05, silica-treated mice compared with same-sex, same-day saline-treated mice. ^†^ *p* < 0.05, silica-treated female mice compared with same-day silica-treated male mice determined by analysis of variance (ANOVA) with Bonferroni’s correction for multiple comparisons.

**Figure 2:**
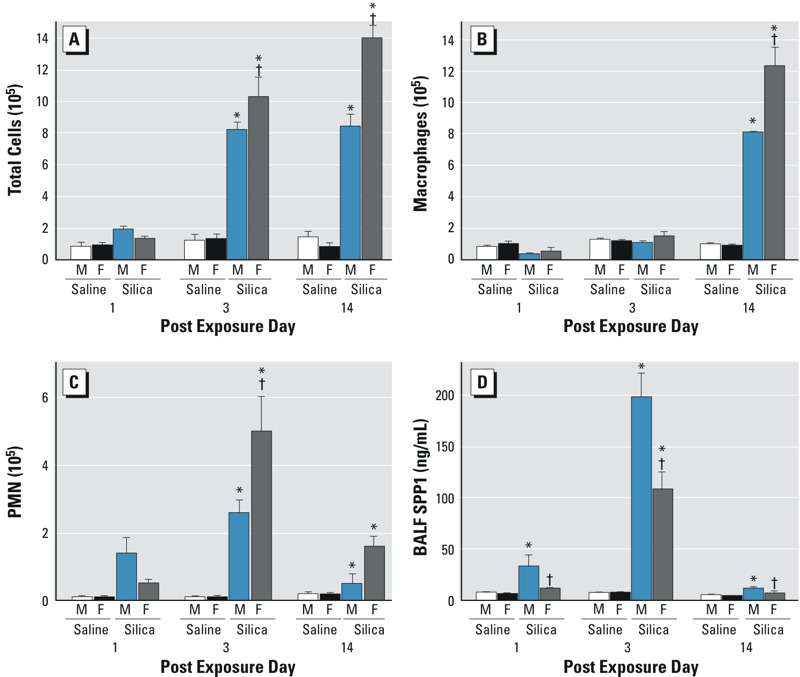
Bronchoalveolar lavage fluid (BALF) cells and secreted phosphoprotein 1 (SPP1) recovered from saline- or silica-treated C57BL/6J mice. (*A*) Total cells at day 3 and day 14, (*B*) macrophages at day 14, and (*C*) neutrophils (PMN) at day 3 increased more in female mice than in male mice following silica exposure. In contrast, (*D*) BALF SPP1 was increased more in silica-exposed male mice than in silica-exposed female mice at days 1, 3, and 14. Values are means ± SE. Saline-treated groups, *n* = 6 mice/sex/day; silica-treated groups, *n* = 5 mice/sex/day. *p < 0.05, silica-treated mice compared with same-sex saline-treated mice. ^†^ *p* < 0.05, silica-treated female mice compared with silica-treated male mice determined by analysis of variance (ANOVA) with Bonferroni’s correction for multiple comparisons.

Increased SPP1 has been associated with pulmonary fibrosis in humans (Foster et al. 2015; Nau et al. 1997; Pardo et al. 2005) and in animal models (Berman et al. 2004; Langley et al. 2011; Ma et al. 2012; Mangum et al. 2004; Miyazaki et al. 1995, Oh et al. 2015; Sabo-Attwood et al. 2011; Takahashi et al. 2001). To determine whether SPP1 is also increased in silica-induced fibrosis, we exposed male and female *Spp1* ^+/+^ mice to crystalline silica and assessed SPP1 levels at 1, 3, and 14 days post-exposure. Silica increased lung SPP1 protein more in male than in female mice (Figure 1). Consistent with other reports (Ganguly et al. 2014; O’Regan 2003), SPP1 protein was detected primarily in macrophages and alveolar epithelial cells in control tissue (Figure 1A). Following silica treatment, SPP1 immunostaining was detected in the extracellular matrix, particularly in males (Figure 1C). Quantitative measurement of immunostaining of SPP1 protein (Figure 1G) and transcripts (Figure 1H) was increased more in males than in females. Analysis of BALF performed after exposure indicated that silica-treated female mice had more total cells at day 3 and 14 (Figure 2A), more neutrophils (PMN) at day 3 (Figure 2C) and more macrophages at day 14 (Figure 2B) than silica-treated male mice. Similar to the SPP1 immunostaining (Figure 1G) and transcripts (Figure 1H), silica-treated male mice had more BALF SPP1 protein than silica-treated female mice (Figure 2D). At day 14, serum SPP1 protein increased more in silica-treated male mice than in silica-treated female mice (male: 175 ± 11 ng/mL, female: 149 ± 7 ng/mL; *p* < 0.05) and in silica-treated mice more than in sex-matched saline-treated mice (male: 116 ± 7 ng/mL, female: 98 ± 4 ng/mL; *p* < 0.05).

### BALF Cells and Protein Recovered from *Spp1* ^+/+^ (C57BL/6J) and *Spp1* ^–/–^ (Gene-Targeted) Mice at 14 Days after Silica Treatment

**Figure 3:**
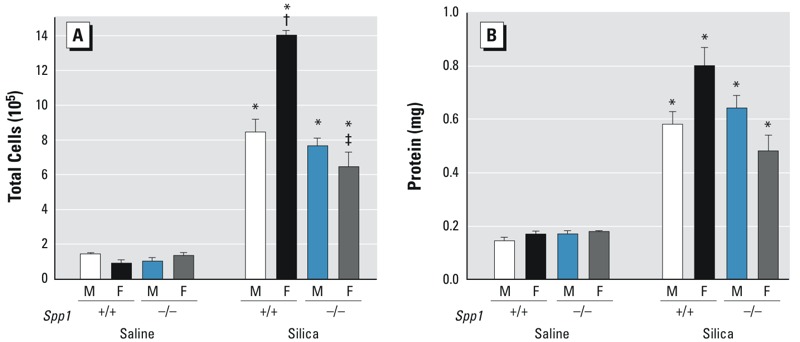
Bronchoalveolar lavage fluid (BALF) cells and protein recovered from saline- or silica-treated *Spp1* ^+/+^ (C57BL/6J) and gene-targeted secreted phosphoprotein 1–null (*Spp1* ^–/–^) mice at 14 days following treatment. (*A*) Fewer BALF total cells were observed in silica-treated *Spp1* ^–/–^ female mice than in control *Spp1* ^+/+^ (C57BL/6J) mice 14 days following silica treatment. (*B*) Although BALF protein in silica-treated mice increased relative to sex- and genotype-matched saline controls, there were no differences between the sexes and the genotypes. Values are means ± SE. Saline-treated groups, *n* = 6 mice/sex/genotype; silica-treated groups, *n* = 6 mice/sex/genotype. *p < 0.05, silica-treated mice compared with same-sex, same-genotype saline-treated mice. ^†^ *p* < 0.05, silica-treated female mice compared with same-genotype silica-treated male mice. ^‡^ *p* < 0.05, silica-treated *Spp1^–/–^* females compared with silica-treated *Spp1* ^+/+^ females determined by analysis of variance (ANOVA) with Bonferroni’s correction for multiple comparisons.

To further define the role of SPP1 in silica-induced lung disease, we treated *Spp1* ^–/–^ mice with silica and assessed inflammatory and fibrotic responses at day 14. BALF from silica-treated mice contained more inflammatory cells than BALF from sex- and genotype-matched saline-exposed mice (Figure 3A). However, silica-exposed *Spp1* ^–/–^ female mice had fewer total BALF cells than silica-exposed *Spp1* ^+/+^ female mice (Figure 3A). Interestingly, the sex difference we observed previously in total cells for *Spp1* ^+/+^ mice (Brass et al. 2010) was not observed in the silica-treated *Spp1* ^–/–^ mice (Figure 3A). No differences in either macrophage or lymphocyte number were observed between silica-exposed male *Spp1* ^–/–^ and silica-exposed female *Spp1* ^–/–^ mice (data not shown). Also consistent with previous observations (Brass et al. 2010), silica exposure increased total BALF protein content (Figure 3B), However, among silica-exposed mice, there were no significant differences in BALF protein between sexes or genotypes.

### Lung Histology, Collagen, and Hydroxyproline in *Spp1* ^+/+^ (C57BL/6J) and *Spp1* ^–/–^ (Gene-Targeted) Mice 14 Days after Silica Treatment

**Figure 4:**
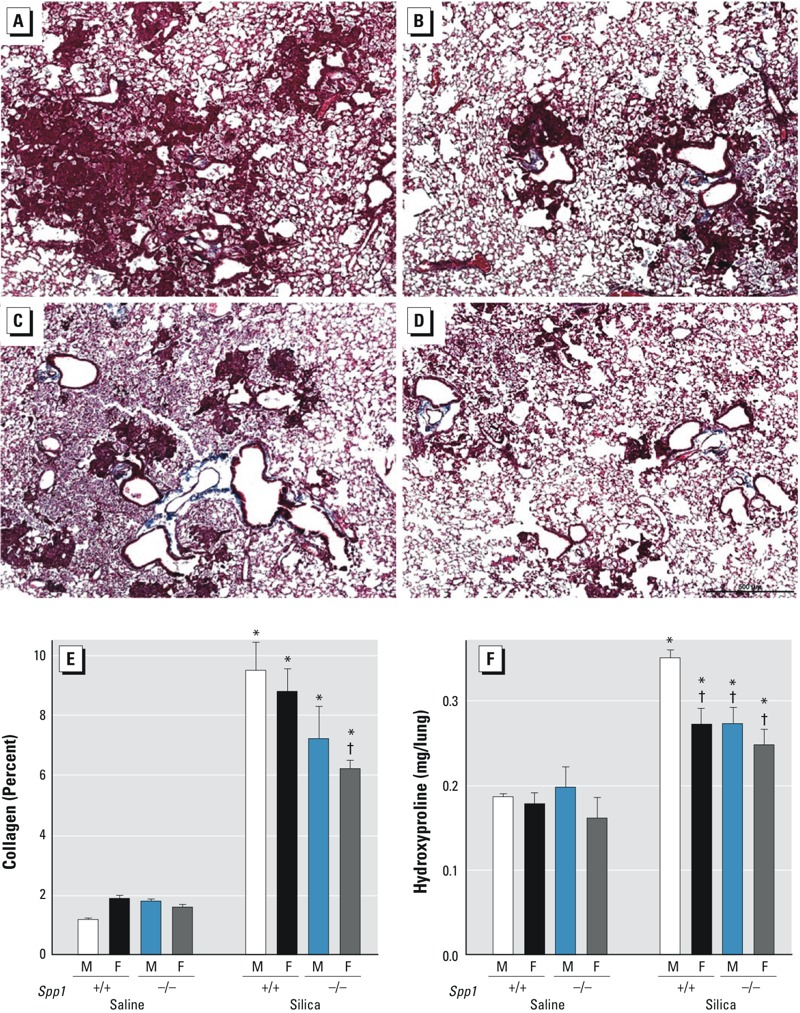
Lung histology, collagen, and hydroxyproline in *Spp1* ^+/+^ (C57BL/6J) and gene-targeted secreted phosphoprotein 1–null (*Spp1* ^–/–^) mice 14 days following silica treatment. Representative section of Masson’s trichrome staining of *Spp1* ^+/+^ mouse lung from silica-exposed (*A*) male and (*B*) female mice (scale bar = 100 μm). Representative sections of Masson’s trichrome staining of *Spp1* ^–/–^ mouse lung from silica-exposed (*C*) male and (*D*) female mice. Lung (*E*) collagen and (*F*) hydroxyproline, an indicator of collagen turnover/deposition, in silica-treated mice at 14 days post-exposure. In silica-exposed male *Spp1* ^+/+^ mice, more (*A*) histological fibrotic lesions and (*F*) hydroxyproline were observed than in silica-exposed (*B* and *F*) *Spp1* ^+/+^ female mice. Smaller fibrotic lesions and less hydroxyproline were also observed in (*C* and *F*) *Spp1* ^–/–^ male and in (*D* and *F*) *Spp1* ^–/–^ female mice than in (*A* and *F*) male *Spp1* ^+/+^ mice. Values are means ± SE. Saline-treated groups, *n* = 6 mice/sex/genotype; silica-treated groups, *n* = 6 mice/sex/genotype. *p < 0.05, silica-treated mice compared with same-sex, same-genotype saline-treated mice. ^†^ *p* < 0.05, silica-treated mice compared with silica-treated *Spp1* ^+/+^ males determined by analysis of variance (ANOVA) with Bonferroni’s correction for multiple comparisons.

In addition to an altered silica-induced BALF total cell response, male (Figure 4C) and female (Figure 4D) *Spp1* ^–/–^ mice had smaller fibrotic lesions than sex-matched male (Figure 4A) and female (Figure 4B) *Spp1* ^+/+^ mice after silica exposure. Similarly, lung hydroxyproline (Figure 4F) decreased in male and female *Spp1* ^–/–^ mice compared with male *Spp1* ^+/+^ mice after silica exposure. Lung hydroxyproline increased more in male *Spp1* ^+/+^ mice than in female *Spp1^+/+^* mice (Figure 4F). No sex difference in lung hydroxyproline was evident between silica-treated male and female *Spp1* ^–/–^ mice. More lung collagen was observed in silica-treated mice than in sex- and genotype-matched saline-treated mice (Figure 4E). Female *Spp1* ^–/–^ mice had less collagen than male *Spp1* ^+/+^ mice after silica exposure.

### SPP1 Protein Expression in Ovariectomized Female and Estrogen-Treated Male Mouse Lung after Silica Treatment

**Figure 5:**
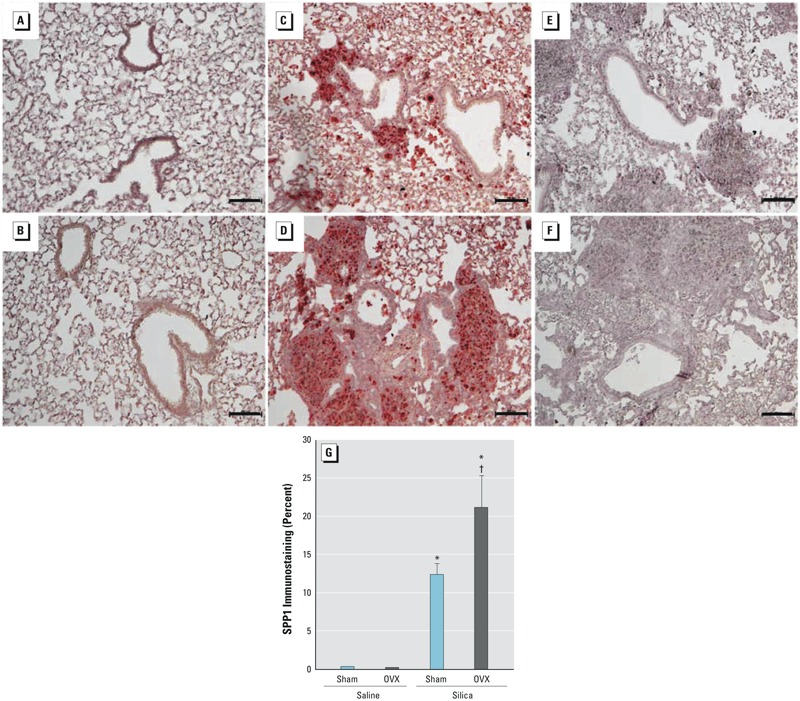
Secreted phosphoprotein 1 (SPP1) expression in sham and ovariectomized (OVX) female C57BL/6 mice 14 days after silica treatment. Bar = 100 μm. Representative lung immunostaining for SPP1 in saline-exposed (*A*) sham and (*B*) OVX female mice and silica-treated (*C*) sham and (*D*) OVX female mice. Representative primary antibody exclusion controls in (*E*) sham and (*F*) OVX female mice 14 days after silica treatment. (*G*) Percentage of tissue SPP1 immuno­staining. Following silica treatment, histological fibrotic lesions and SPP1 immunostaining increased more in OVX mice than in sham female mice. Saline-treated groups, *n* = 6 mice/sex/day; silica-treated groups, *n* = 6 mice/sex/day. *p < 0.05, silica-treated mice compared with group-matched saline-treated mice. ^†^ *p* < 0.05, silica-treated OVX mice compared with silica-treated sham mice determined by analysis of variance (ANOVA) with Bonferroni’s correction for multiple comparisons.

Based on the above-mentioned observations and on our previous work (Brass et al. 2010), we hypothesized that the protection against silica-induced fibrosis observed in females could be caused, in part, by estrogen-mediated changes in SPP1 expression. Ovariectomized female mice showed increased SPP1 immunostaining in the lung (Figure 5D,G) compared with sham female mice (Figure 5C,G), suggesting that estrogen suppressed the activation of SPP1 levels.

**Figure 6:**
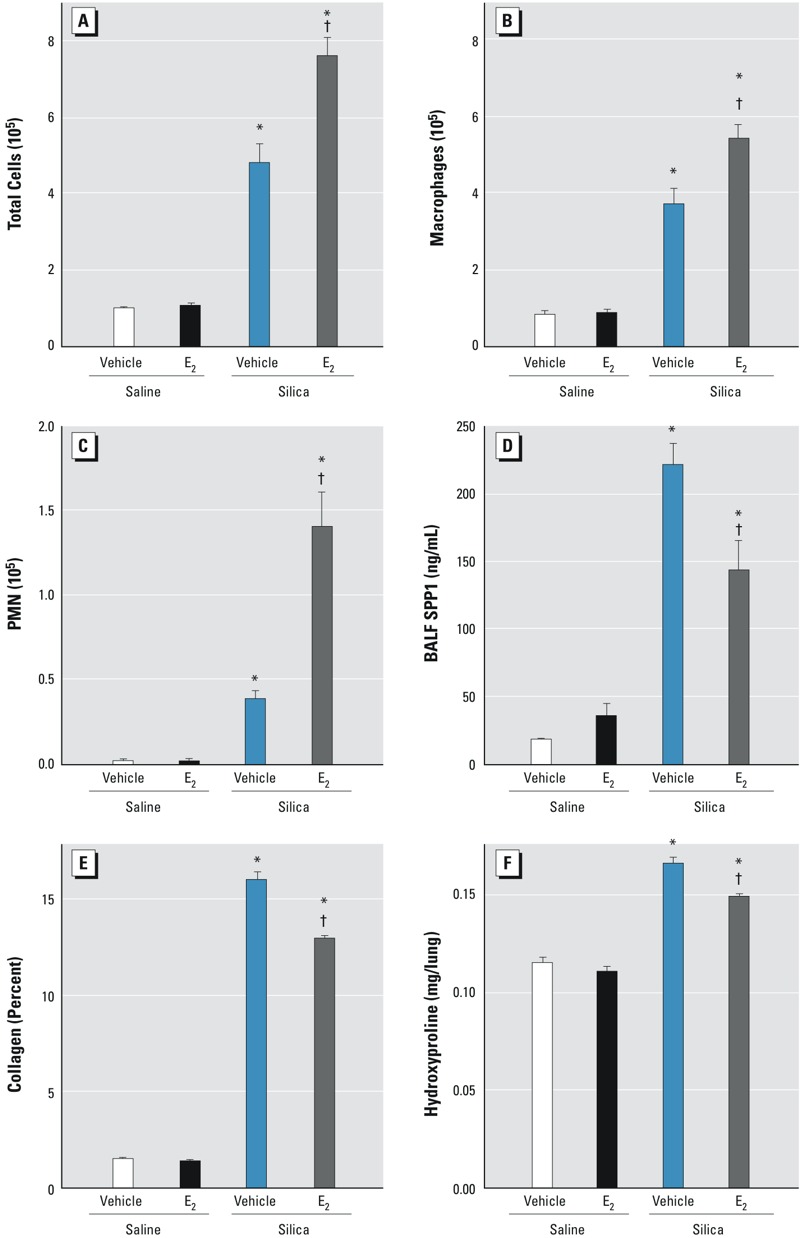
Bronchoalveolar lavage fluid (BALF) cells and secreted phosphoprotein 1 (SPP1) in vehicle-treated male and estrogen-treated (E_2_) male C57BL/6J mice 14 days after silica treatment. Silica-induced BALF (*A*) total cells, (*B*) macrophages, and (*C*) neutrophils (PMN) increased, and (*D*) SPP1 protein decreased with estrogen treatment. Lung (*E*) collagen and (*F*) hydroxyproline were decreased by estrogen treatment. Values are means ± SE. Saline-treated groups, *n* = 6 mice/sex/day; silica-treated groups, *n* = 5 mice/sex/day. *p < 0.05, silica plus vehicle group versus saline plus vehicle group or silica plus estrogen group versus saline plus estrogen group*.* ^†^ *p* < 0.05, silica plus estrogen group versus silica plus vehicle group determined by analysis of variance (ANOVA) followed by Bonferroni’s correction for multiple comparisons.

To confirm the role of estrogen in determining SPP1 levels and silica-induced lung fibrosis, we pretreated male mice with estrogen for 21 days prior to silica administration. Following exposure to silica, estrogen pretreatment resulted in increased BALF total cells, macrophages, and PMN (Figure 6A–C); decreased BALF SPP1 (Figure 6D); and decreased lung collagen deposition (Figure 6E) and hydroxyproline (Figure 6F). These changes mirrored the increased BALF total cells (Figure 2A) and decreased BALF SPP1 (Figure 2D), collagen deposition, and hydroxyproline (Figures 1–4) observed in female mice when compared with males. Together, these findings support the concept that estrogen is protective against the development of pulmonary fibrosis, perhaps though the recruitment of additional phagocytic inflammatory cells.

## Discussion

In this study, we hypothesized that differences in sensitivity to silica-induced pulmonary fibrosis between male and female mice are caused, in part, by alterations in the proinflammatory and profibrotic protein SPP1. After silica exposure, SPP1 increased to a greater degree in the lung tissue, BALF, and serum of male mice than in those of female mice. Additionally, SPP1 is likely to be a pro-fibrotic mediator inasmuch as *Spp1* ^–/–^ male mice were resistant to silica-induced fibrotic lesions (Figure 4C) and hydroxyproline (Figure 4F) compared with *Spp1* ^+/+^ male mice (Figure 4A,F). SPP1 appears to be a sex-specific mediator because *a*) the sex differences in silica sensitivity were not evident in exposed *Spp1* ^–/–^ mice; *b*) silica-induced increases in SPP1 were greater in ovariectomized mice than in sham female mice; and *c*) the silica-induced increases in SPP1, collagen, and hydroxyproline were smaller in male mice pretreated with estrogen than in untreated male mice. Taken together, these data suggest that estrogen-mediated repression of SPP1 levels accounts for, at least in part, the relative resistance of female mice to silica-induced fibrosis.

SPP1 contributes to human fibrotic lung disease, and increases in SPP1 are associated with pulmonary fibrosis (Foster et al. 2015; Nau et al. 1997; Pardo et al. 2005) and with the development of fibrosis in a number of animal models (Berman et al. 2004; Langley et al. 2011; Ma et al. 2012; Mangum et al. 2004; Miyazaki et al. 1995; Oh et al. 2015; Sabo-Attwood et al. 2011; Takahashi et al. 2001). For example, bleomycin treatment of mice results in an increase in SPP1 mRNA and protein at 14 days post-exposure, and treatment with an antibody that blocks SPP1 function partially protects against fibrosis development (Takahashi et al. 2001). Here, SPP1 protein levels are increased in lung tissue, BALF, and serum in response to silica exposure in both male and female *Spp1* ^+/+^ mice. We also observed more SPP1 mRNA at 3 and 14 days post-exposure in silica-treated mice than in saline-treated controls (Figure 1H). Notably, increased SPP1 mRNA has been reported in tumor necrosis factor (TNF)-induced lung fibrosis in mice (Miyazaki et al. 1995), which may be of particular importance in the pathogenesis of silica-induced lung disease because lung TNF is increased in silicosis patients (Vanhée et al. 1995) and in silica-treated mice (Ortiz et al. 1998).

SPP1 is often described as a matricellular protein, indicating that it is localized to both the extracellular matrix and the cell cytoplasm (O’Regan 2003; Wang and Denhardt 2008). Consistent with previous reports (Takahashi et al. 2001), silica-induced SPP1 immunostaining increased in resident alveolar macrophages and alveolar epithelial cells (Figure 1C–E). These cells express both intracellular and secreted SPP1 (Ganguly et al. 2014; O’Regan 2003; Takahashi et al. 2001; Wang and Denhardt 2008). Secreted SPP1 present in lung tissue (Figure 1G) and BALF (Figure 2D) of silica-treated male mice increased more than that in silica-treated female mice. In addition, BALF SPP1 did not increase significantly in silica-treated versus saline-treated female mice on days 1 and 14 and was only evident on day 3. In response to tissue injury, secreted SPP1 mediates the migration, proliferation, adhesion, and differentiation of fibroblasts and myofibroblasts (Lenga et al. 2008). Secreted SPP1 is also required for the proper deposition and organization of the extracellular matrix during tissue repair processes. This requirement has been noted in a variety of fibrosis models, including bleomycin-induced fibrosis, and *Spp1* ^–/–^ mice had reduced collagen deposition and disorganized collagen fibrils at sites of injury (Berman et al. 2004). Consistent with these observations, lung hydroxyproline content was lower in the *Spp1* ^–/–^ male mice than in the *Spp1^+/+^* male mice (Figure 4F). Recently, we reported that SPP1 promotes pneumocyte growth and that *Spp1^–/–^* mice have smaller, more compliant lungs with enlarged airspace [i.e., increased mean airspace chord length (*L*m)] (Ganguly et al. 2014). Taken together, these findings from previous studies and our own data suggest that lung SPP1 could contribute to fibrosis and that the sex-specific sensitivity may be due to differing levels of secreted SPP1 present in male and female lungs.

SPP1 is a known component of several fibrosis-associated pathophysiologic pathways. For example, TGFB1 is an essential mediator of pulmonary fibrosis and can induce SPP1 expression. Conversely, fibroblasts from *Spp1* ^–/–^ mice are insensitive to stimulation by TGFB1, indicating that SPP1 is an essential component of TGFB1 signaling in these cells (Mori et al. 2008). SPP1 contains a number of functional domains including a canonical RGD integrin-binding domain and can interact with various integrins (Sodek et al. 2000). SPP1-integrin binding induces the assembly of actin filaments in the cytoplasm, resulting in increased cell and tissue stiffness and leading to the mechanosensitive activation of TGFB1 (Henderson and Sheppard 2013). In addition, SPP1 can bind to heteromeric integrin αvβ6 (Erikson et al. 2009), the primary TGFB1-binding integrin (Munger et al. 1999; Sheppard 2015); SPP1 may compete with TGFB1 for integrin αvβ6 binding and thus stimulate many of the same cellular responses as TGFB1.

Our silica model has an important inflammatory component, in that female mice have more total cells (Figure 2A on days 3 and 14), macrophages (Figure 2B on day 14), and neutrophils (Figure 2C on days 3 and 14) than male mice following silica exposure. The role of inflammation in pulmonary fibrosis has been controversial for many years, and it remains unclear whether antiinflammatory therapy could be effective in treating fibrosis (Noble and Homer 2005). In the present study, inflammation appears to be inversely related to the fibrotic response, suggesting that the severity of the inflammatory response may not predict the degree of fibrotic response in this model system. This observation may be influenced by the limitations of murine models. Nonetheless, animal models have been useful in uncovering specific profibrotic molecular mechanisms and pathways (Barkauskas and Noble 2014; Moore et al. 2013). The roles of macrophage and epithelial cell activation and of elicited growth factors, cytokines, and other proteins typically associated with inflammation are still considered to be important in the etiology of fibrosis (Wolters et al. 2014). Our present observations support the notion that the role of inflammatory cells is complex and that they may both potentiate and resolve fibrosis.

Macrophages are thought to be the key inflammatory cells that mediate silica-induced lung injury (Leung et al. 2012). Macrophages also express high levels of SPP1, and SPP1 is thought to mediate macrophage recruitment and activation though both extracellular and intracellular pathways (Rittling 2011). Phagocytosis of particles by macrophages is the first line of defense against the inhalation of silica (or other particulates), and SPP1 can alter phagocytosis of bacteria and bacterial particles (Rittling 2011). As noted previously (Brass et al. 2010), female mice that had less fibrosis than males in response to silica exposure also had more macrophages in their lung lavage fluid. The combination of increased numbers of macrophages and decreased SPP1 (Figures 1 and 2) may result in greater clearance of silica particles from the lungs of female mice than from those of males.

Exactly how female sex influences SPP1 expression remains unknown. One possibility is that sex-specific expression of SPP1 has a genetic basis. Several genetic variants in the human *SPP1* promoter have been identified and are functional (Giacopelli et al. 2004). One single-nucleotide polymorphism (SNP), rs28357094, is associated with decreased SPP1 expression (Giacopelli et al. 2004). Interestingly, this SNP has been associated with sex-related effects in disease severity in systemic lupus erythematosus (Kariuki et al. 2009) and juvenile dermatomyositis (Niewold et al. 2010). Although several genome-wide association studies have been conducted to identify SNPs associated with the development and progression of pulmonary fibrosis (Fingerlin et al. 2013; Mushiroda et al. 2008; Noth et al. 2013; Seibold et al. 2011), differences in SNP associations between the sexes were not examined in either affected patients or controls. It is likely that the presence of a protective sex-specific SNP may have gone undetected.

Whether estrogen plays a direct or an indirect role in determining sex-specific expression of SPP1 is another unanswered question. In other organ systems, *SPP1* promoter activation is estrogen-sensitive, and estrogen can both stimulate (Vanacker et al. 1998, 1999) and inhibit (Arias-Loza et al. 2007; Li et al. 2000) SPP1 production depending on the cell type involved. In vascular endothelial cells, estrogen can modulate *SPP1* transcription through a nuclear factor (NF)-κB–mediated mechanism (Lund et al. 2009; Wang and Denhardt 2008), specifically by facilitating the formation of either transcription-activating or transcription-repressing NF-κB dimers (Urtasun et al. 2012). Therefore, it is possible that estrogen indirectly represses *Spp1* transcription through NF-κB in female mice and that the lack of estrogen-mediated repression allows the excessive production of SPP1 in male mice.

## Conclusion

In summary, we previously reported a sex-dependent response to silica-induced pulmonary fibrosis in mice (Brass et al. 2010). Here, we have identified SPP1 as being differentially expressed in an estrogen-dependent manner and contributing, in part, to the observed differences in silica-induced fibrosis between male and female mice. The relationship of sex to disease susceptibility and development is complex and is in need of further exploration. Determining how such a fundamental characteristic as sex influences disease development and outcome is essential to realizing the goals of stratified medicine.
